# Exometabolome and Molecular Signatures Associated with HPV 16 in Cervical Cancer: Integrative Metabolomic and Transcriptomic Analysis for Biomarker Discovery

**DOI:** 10.3390/molecules30193909

**Published:** 2025-09-28

**Authors:** Adán Arizmendi-Izazaga, Napoleón Navarro-Tito, Gabriela Elizabeth Campos-Viguri, Hilda Jiménez-Wences, Macdiel Emilio Acevedo-Quiroz, Eric Genaro Salmerón-Bárcenas, Berenice Illades-Aguiar, Marco Antonio Leyva-Vázquez, Julio Ortiz-Ortiz

**Affiliations:** 1 Laboratorio de Investigación en Metabolismo y Cáncer, Facultad de Ciencias Químico Biológicas, Universidad Autónoma de Guerrero, Av. Lázaro Cárdenas S/N, Ciudad Universitaria, Colonia La Haciendita, Chilpancingo 39090, Guerrero, Mexico; adanarizmendi@uagro.mx (A.A.-I.); wences2009@hotmail.com (H.J.-W.); 2 Laboratorio de Biología Celular del Cáncer, Facultad de Ciencias Químico Biológicas, Universidad Autónoma de Guerrero, Av. Lázaro Cárdenas S/N, Ciudad Universitaria, Colonia La Haciendita, Chilpancingo 39090, Guerrero, Mexico; nnavarro@uagro.mx; 3 Centro de Investigación en Ciencia Aplicada y Tecnología Avanzada Unidad Morelos, Instituto Politécnico Nacional, Boulevard de la Tecnología, 1036 Z-1, P 2/2, Atlacholoaya 62790, Morelos, Mexico; gabyirugiv@gmail.com; 4 Laboratorio de Investigación en Biomoléculas, Facultad de Ciencias Químico Biológicas, Universidad Autónoma de Guerrero, Av. Lázaro Cárdenas S/N, Ciudad Universitaria, Colonia La Haciendita, Chilpancingo 39090, Guerrero, Mexico; 5 Laboratorio de Investigación en Enfermedades Infecciosas y Cáncer, Facultad de Ciencias Químico Biológicas, Universidad Autónoma de Guerrero, Av. Lázaro Cárdenas S/N, Ciudad Universitaria, Colonia La Haciendita, Chilpancingo 39090, Guerrero, Mexico; 6 Department of Chemical and Biochemical Engineering, National Technological Institute of Mexico, Technological/IT Institute of Zacatepec, Zacatepec 62780, Morelos, Mexico; macdiel.aq@zacatepec.tecnm.mx; 7 Departamento de Biomedicina Molecular, Centro de Investigación y de Estudios Avanzados del Instituto Politécnico Nacional, Ciudad de México 07360, Mexico; eric.salmeron@cinvestav.mx; 8 Laboratorio de Biomedicina Molecular, Facultad de Ciencias Químico Biológicas, Universidad Autónoma de Guerrero, Av. Lázaro Cárdenas S/N, Ciudad Universitaria, Colonia La Haciendita, Chilpancingo 39090, Guerrero, Mexico; b.illadesaguiar@gmail.com (B.I.-A.); leyvamarco13@gmail.com (M.A.L.-V.)

**Keywords:** cervical cancer, human papillomavirus 16 (HPV 16), metabolic reprogramming, exometabolome, transcriptomics, ^1^H-NMR, molecular signatures, biomarkers

## Abstract

Cervical cancer (CC) represents a major public health concern, ranking as the fourth most frequently diagnosed cancer and one of the leading causes of cancer-related mortality among middle-aged women worldwide. CC is caused by persistent infection with high-risk human papillomaviruses (HR-HPVs), with HPV 16 being the cause of more than 50% of CC cases. In this study, the exometabolome of the HPV 16-positive cell lines SiHa and Ca Ski, as well as the HPV 16-negative control cell line C-33 A, was evaluated. The exometabolome was validated through molecular signatures using a transcriptomic approach to identify genes encoding cellular metabolic enzymes. The exometabolome was analyzed using ^1^H nuclear magnetic resonance spectroscopy (^1^H-NMR). Exometabolomic profiles were subsequently compared through both multivariate and univariate statistical analyses to identify significant differences between cell lines. Molecular signatures were analyzed from the GSE9750 dataset obtained from the GEO database. Exometabolic profiling of the HPV 16 positive cell lines showed higher concentrations of leucine, isoleucine, valine, lysine, methionine, glutamine, ornithine, choline, glucose, and tryptophan. An expression analysis showed increased expression of enzymes involved in amino acid synthesis, the tricarboxylic acid cycle, glycolysis, the pentose phosphate pathway, galactose metabolism, and HIF-1α. These data suggest metabolites and metabolism-associated genes that can be used as non-invasive, stable diagnostic and prognostic biomarkers, as well as therapeutic targets for CC in the presence of HPV 16.

## 1. Introduction

Cervical cancer (CC) is the fourth most common cancer and the leading cause of cancer-related death in middle-aged women [1,2]. Precursor lesions and progression to CC are caused by persistent infection with high-risk human papillomavirus (HR-HPV), and HPV 16 is the most common [3]. In this regard, the expression of the viral oncoproteins E6 and E7 of HPV 16 are involved in cell transformation and tumor generation [4,5,6]. However, in addition to virus-associated factors such as continuous expression of viral oncogenes E6 and E7, E2 gene cleavage and integration of viral DNA into host cell DNA, viral load and genetic variability, host-associated factors such as immunological, genetic, epigenetic, and metabolic factors are required for tumor development and progression [3,7,8].

Cancer cells have been shown to adopt different metabolic patterns compared with normal cells [9]. Cancer cells consume more glucose to obtain sufficient energy through glycolysis, even in the presence of oxygen, a process known as the Warburg effect [10], a redox state that provides proteins, nucleic acids, lipids, and other macromolecules [9,11,12]. In addition to different types of cancer, metabolic patterns have been identified in CC that may serve as biomarkers of progression, including the dysregulation of glycolytic metabolism, high lactate levels, decreased α- and β-glucose levels, increased low-density lipoproteins levels, lipid accumulation, and abnormal kynurenine/tryptophan ratios [13,14]. E6 and E7 from HPV 16 have been proposed to participate in metabolic reprogramming by regulating proteins responsible for the degradation of hypoxia-inducible factor 1α (HIF-1α) [15]. E6 and E7 can interact with VHL, CUL2, and ELOC, inhibiting the formation of the E3 ubiquitin ligase complex that ubiquitinates HIF-1α for subsequent degradation via the proteasomal pathway. On one hand, E6 interacts with VHL by blocking its interaction with HIF-1α; on the other hand, E7 interacts with CUL2 and ELOC, preventing their binding to VHL and RBX1, respectively. Consequently, HIF-1α stabilizes and binds with HIF-1β to form the active HIF1 complex, which binds to hypoxia response elements (HREs) in DNA, allowing for the expression of genes related to energy metabolism such as glucose transporter member 1 (*GLUT1*), glycolytic enzymes such as hexokinase II (*HKII*), phosphofructokinase (*PFK*), enolase A (*ENOA*), pyruvate kinase M2 (*PKM2*) and lactate dehydrogenase A (*LDHA*) [15,16]. In contrast, the mitochondrial oxidative phosphorylation system (OXPHOS) is affected by HPV 16 at the level of the mitochondrial structure by the release of and increase in reactive oxygen species (ROS), the inactivation of mitochondrial complex III and ATP synthase, and decreased levels of glutathione (GSH) and superoxide dismutase 1 and 2 (SOD1 and SOD2) [16]. Additional mechanisms by which E6 promotes metabolic reprogramming involve the deregulation of proteins and signaling pathways, including p53, c-Myc, and STAT3 and the Wnt/β-catenin, MAPK, mTOR, and PI3K/AKT pathways. Specifically, E7 promotes metabolic reprogramming by inactivating the retinoblastoma protein [17].

At present, poor prognosis and absence of symptoms in the early stages hinder early diagnosis of gynecological cancers such as CC. Thus, prompt intervention is required to improve the survival statistics of cancer patients and reduce the social and financial burdens of the disease [18]. Currently employed conventional screening and cytological diagnostic methods have low sensitivity and specificity, and are not widely used for risk assessment, resulting in delayed diagnosis of CC. In addition, metabolomics-focused studies are a current resource that focuses on the analysis of endpoint metabolites; specifically, the end products generated or maintained by genetic or environmental changes in a living system. The metabolome then provides an identification profile of the cell end condition, discriminates tumor phenotype, and is an efficient platform to differentiate between healthy individuals and cancer patients [18]. In addition, metabolomics can be applied in in vitro systems; for example, in exometabolomics studies for the detection of changes in the composition of cell growth media [19]. Exometabolomics offers a powerful strategy for identifying cellular phenotypes while avoiding the challenges of measuring intracellular metabolites because exometabolite levels are integrated within minutes or hours, whereas intracellular metabolites can be altered or degraded within milliseconds of extraction [19]. Recently, through a study of the CC metabolome, new potential non-invasive markers have been identified to predict the disease substage [20].

This work aims to analyze the exometabolome of three cell models positive and negative to HPV 16. We performed a ^1^H nuclear magnetic resonance (^1^H-NMR) metabolomic profile analysis of the culture media used by HPV 16 positive and negative CC cell lines to identify potential biomarkers for the diagnosis and progression of CC. In addition, using a transcriptomic approach, we validated and correlated the expression of metabolic enzymes that are underexpressed in HPV 16-positive and negative CC cell lines. This global and specific approach could be useful in discovering new biomarkers for disease risk prediction.

## 2. Results

### 2.1. Unsupervised Analysis of Culture Media Reveals HPV 16–Driven Metabolic Discrimination in Cervical Cancer Cell Lines

**Figure 1:**
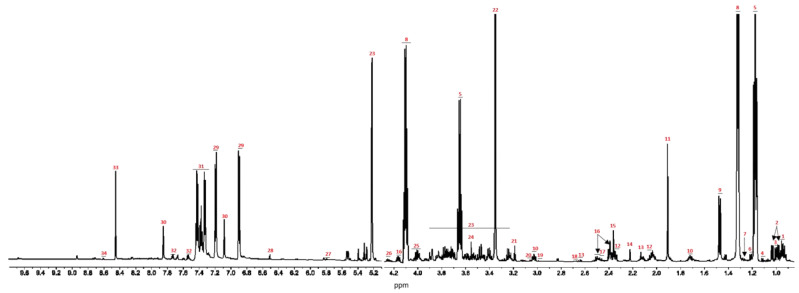
Assignment of metabolites in a typical H-CPMG spectrum of culture medium. The water signal has been excluded from the spectrum. The region of the spectrum between 5 and 9 ppm has been expanded. The assignment of metabolites is: (1) Leucine (left 0.97 ppm and right 0.94 ppm); (2) Valine (left 0.99, 1.05 ppm and right 0.97, 1.01 ppm); (3) Isoleucine (left 1.01 ppm and right 0.99 ppm); (4) Propylene glycol (left 1.12 ppm and right 1.10 ppm); (5) Ethanol (left 1.20, 3.68 ppm and right 1.17, 3.62 ppm); (6) 3 Hydroxybuyrate (left 1.22 ppm and right 1.20 ppm); (7) 3-hydroxyisovalerate (left 1.28 ppm and right 1.27 ppm); (8) Lactate (left 1.34, 4.13 ppm and right 1.29, 4.07 ppm); (9) Alanine (left 1.50 ppm and right 1.45 ppm); (10) Lysine (left 1.75, 3.03 ppm and right 1.70, 3.01 ppm); (11) Acetate (left 1.93 ppm and right 1.89 ppm); (12) Glutamate (left 2.08, 2.35 ppm and right 2.04, 2.32 ppm); (13) Methionine (left 2.14, 2.66 ppm and right 2.12, 2.62 ppm); (14) Acetone (left 2.23 ppm and right 2.21 ppm); (15) Pyruvate (left 2.37 ppm and right 2.35 ppm); (16) Pyroglutamate (left 2.39, 2.53, 4.19 ppm and right 2.37, 2.47, 4.14 ppm); (17) Glutamine (left 2.45 ppm and right 2.43 ppm); (18) Citrate (left 2.67 ppm and right 2.66 ppm); (19) 2-oxoglutarate (left 2.99 ppm and right 2.97 ppm); (20) Ornithine (left 3.05 ppm and right 3.04 ppm); (21) Choline (left 3.19 ppm and right 3.18 ppm); (22) Methanol (left 3.37 ppm and right 3.32 ppm); (23) Glucose (left 3.91, 5.24 ppm and right 3.21, 5.19 ppm); (24) Glycine (left 3.56 ppm and right 3.55 ppmc; (25) Isopropanol (left 4.04 ppm and right 3.99 ppm); (26) Threonine (left 4.27 ppm and right 4.23 ppm); (27) Uracil (left 5.81 ppm and right 5.78 ppm); (28) Fumarate (left 6.52 ppm and right 6.49 ppm); (29) Tyrosine (left 6.93, 7.22 ppm and right 6.86, 7.15 ppm); (30) Histidine (left 7.11, 7.87 ppm and right 7.05, 7.82 ppm); (31) Phenylalanine (left 7.47 ppm and right 7.30 ppm); (32) Tryptophan (left 7.55, 7.75 ppm and right 7.52, 7.70 ppm); (33) Formate (left 8.48 ppm and right 8.42 ppm); (34) Nicotinate (left 8.62 ppm and right 8.58 ppm).

**Figure 2:**
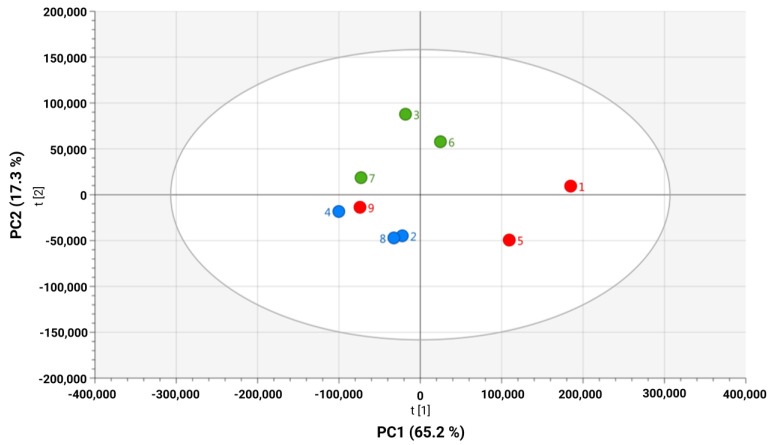
Principal component analysis (PCA) score plot off medium global metabolic profile of SiHa (red ●), Ca Ski (blue ●), and C-33 A (green ●) cell lines. Principal Component 1 (PC1 65.2%) is plotted vs. Principal Component 2 (PC2 17.3%).

To evaluate the possible influence of HPV 16 on the metabolic profiles obtained from the C-33 A, SiHa, and Ca Ski cell lines, an unsupervised analysis of ^1^H-NMR spectra was performed (Figure 1). Differences in the clustering of the data were observed when comparing the three cell models (Figure 2). A Principal Component Analysis (PCA) revealed a clear, high variation (82.5%) between the C-33 A, SiHa, and Ca Ski cell lines, with PC1 contributing 65.2% of the variation. The unsupervised data reveal differences between the datasets in the C-33 A, SiHa and Ca Ski cell lines, suggesting that the presence of HPV 16 and the integrated copy number contribute to alterations in the different metabolomic profiles of the media where the cell lines were cultured.

### 2.2. Supervised Analysis Reveals Significant Exometabolomic Differences Between HPV 16-Positive and -Negative Cervical Cancer Cell Lines

**Figure 3:**
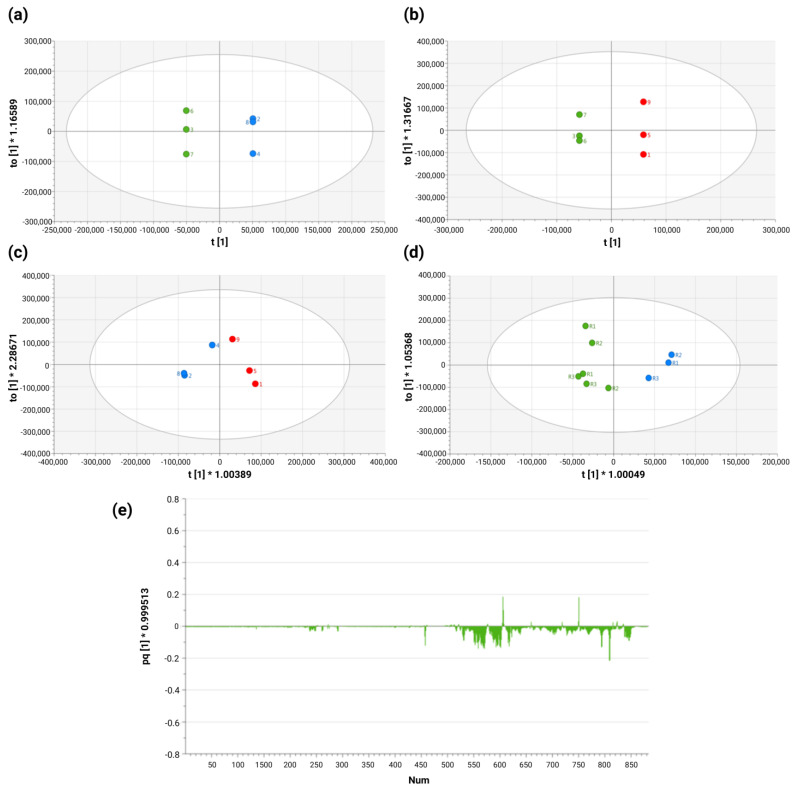
Orthogonal Partial Least Squared Discriminant Analysis (OPLS-DA) score plots for the comparison between: (**a**) C-33 A vs. Ca Ski, (**b**) C-33 A vs. SiHa and (**c**) Ca Ski vs. SiHa. In panels (**a**–**c**), the green color (●) represents the C-33 A cell line, in blue color (●) the Ca Ski cell line, and in red color (●) the SiHa cell line. (**d**) OPLS-DA score plots of comparison between HPV 16-positive (green ●) and HPV 16-negative (blue ●) cells. (**e**) Loading plot of OPLS-DA score plot.

To determine the differences between the exometabolome of HPV 16-positive and -negative cell lines, statistical discriminant models (OPLS-DA) were utilized based on comparisons between the three different groups of cells included in the training set (Figure 3), with the significance of the OPLS-DA model being assessed using a cross-validation ANOVA (*p* ≤ 0.05 was considered significant) and a permutation test (n = 100). In the analysis, C-33 A, SiHa, and Ca Ski cells were found to exhibit a specific metabolic profile (R^2^X = 1; Q^2^ = 1) characterized by statistically significant differences in the concentrations of several metabolites (Figure 3a,b). We observed that in the SiHa and Ca Ski cell lines, which exhibit variations in the number of HPV 16 copies integrated into their genomes, specific metabolic profiles were present, and had statistically significant differences in the concentrations of various metabolites of R^2^X = 0.834; Q^2^ = 0.159 (Figure 3c). When the comparison was made between the HPV 16-negative and -positive groups, we observed that the exometabolomic profile is different and specific for HPV 16-negative cells compared with the HPV 16-positive cell lines, in which the presence and viral load alter the exometabolome and the viral load exhibits a significant effect, yielding statistically significant differences in the concentrations of various metabolites with R^2^X = 0.925; Q^2^ = 0.749 (Figure 3d). In a subset of highly discriminating metabolites between the two analyzed groups, marked differences were observed in intensity, which are considered responsible for the separation observed in the OPLS-DA space (Figure 3e).

**Figure 4:**
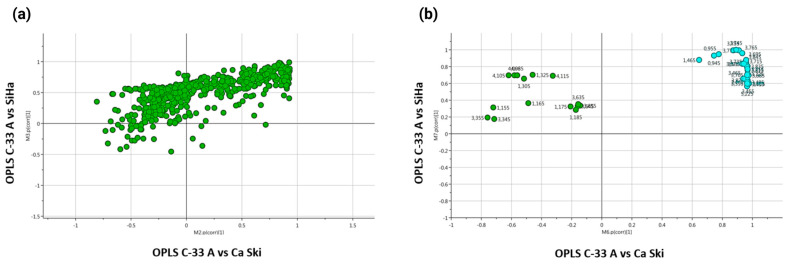
SUS-plot graphs of comparison of OPLS-DA models of the negative HPV 16 cell line (C-33 A cells) and both positive HPV 16 cell lines (SiHa and Ca Ski). (**a**) SUS-plot plots of comparison of OPLS-DA models between C-33 A vs. Ca Ski and C-33 A vs. SiHa. (**b**) SUS-plot between C-33 A vs. Ca Ski and C-33 A vs. SiHa considering only the metabolites with a VIP greater than 1.5 and are common between both models.

An analysis of metabolic differences based on the results of the shared and single structure plots (SUS-plots) (Figure 4) revealed significant common variations between the metabolic profile of the exometabolome of the HPV 16-negative cell line (C-33 A cells) and both positive cell lines (SiHa and Ca Ski cells) (Figure 4a,b). The blue dots in Figure 4b represent metabolites whose variation is in the same direction in both models and have a VIP greater than 1.5 in the individual models. The common metabolites in both OPLS were alanine, isoleucine, leucine, glutamate–glutamine, trimethylamine-n-oxide, and glucose.

### 2.3. Specific Combinations of Metabolites Discriminate HPV 16-Negative and Positive Cells

To determine the differential profile of the exometabolites of the C-33 A cell lines (HPV 16-negative) and SiHa and Ca Ski cells (HPV 16-positive), an inspection of the contribution to the separation between groups was performed, which resulted in the identification of the spectral signals and metabolites that contributed the most to the discrimination between the groups being compared. Using this approach, the concentrations of 11 metabolites showed statistically significant differences when compared between the study groups (Table 1). When the metabolite concentrations of the SiHa and Ca Ski cells were compared, significant differences were observed in glutamine. When comparing the C-33 A cells with the SiHa cells, significant differences were observed in glutamine and uracil. Furthermore, the glucose concentration showed statistically significant differences compared with the C-33 A cell for the Ca Ski cells. To determine the differential exometabolite profile of the C-33 A cells (HPV 16-negative) and the SiHa and Ca Ski cells (HPV 16-positive), their contributions to group separation were analyzed, allowing for the identification of spectral signals and metabolites. Leucine, isoleucine, valine, lysine, methionine, glutamine, ornithine, choline, glucose, and tryptophan were found to show significant differences when comparing the exometabolome of HPV 16-positive and -negative cell lines. These data confirm significant differences between the exometabolic profiles of HPV 16-negative (C-33 A) and -positive (SiHa and Ca Ski) cancer cell. These results could provide essential data for the diagnosis and prognosis of CC.

**Figure 5:**
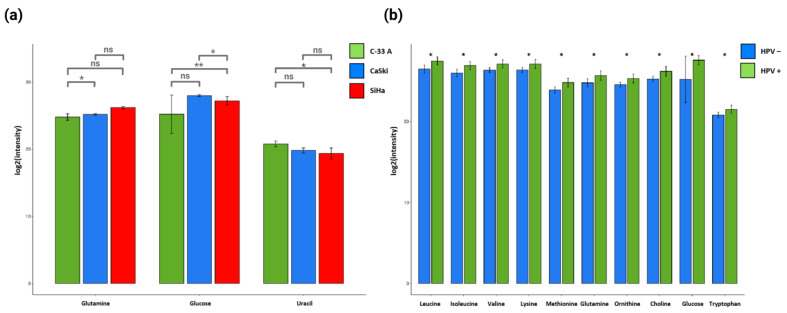
Concentration of metabolites in the C-33 A, SiHa, and Ca Ski cell lines. The logarithmic bar graph in (**a**) represents the intensity of the metabolites in the three cell groups: C-33 A (green bars), Ca Ski (blue bars), and SiHa (red bars). The logarithmic bar graph in (**b**) represents the intensity of the metabolites when comparing HPV 16 positive (SiHa and Ca Ski) and negative (C-33 A) cell lines. The intensity of the metabolites was analyzed in GraphPad Prism 8 software using Student’s *t* test. ns (not significant). Statistical significance: * *p* < 0.05, ** *p* < 0.01.

Metabolite levels were analyzed in depth, revealing significant differences in the metabolic profiles of HPV 16-negative and -positive cells (Figure 5). The concentration of glutamine was found to be higher in the SiHa cell line, while glucose showed high concentrations in the Ca Ski cell line, but uracil concentrations were higher in the C-33 A cell line (Figure 5a). When comparing the concentrations of metabolites in HPV 16-positive and -negative cell lines, the concentrations of leucine, isoleucine, valine, lysine, methionine, glutamine, ornithine, choline, glucose, and tryptophan were observed to be significantly higher in the Ca Ski cells (with a higher number of HPV 16 DNA copies integrated into the cell genome) and SiHa cells (with a lower number of HPV 16 DNA copies integrated into their genome) and lower in the C-33 A cells (negative cancer cells to HPV 16) (Figure 5). These data support the hypothesis that the presence of HPV 16; the density of its DNA integration into the cell genome; and, therefore, the viral load in CC cells are related to specific exometabolomic profiles. These findings may be helpful in the non-invasive identification of biomarkers associated with the diagnosis and progression of CC.

### 2.4. The Exometabolome Is Associated with the Expression of Genes Related to Metabolic Reprogramming in HPV 16-Positive and -Negative Cervical Cancer Cell Lines

**Figure 6:**
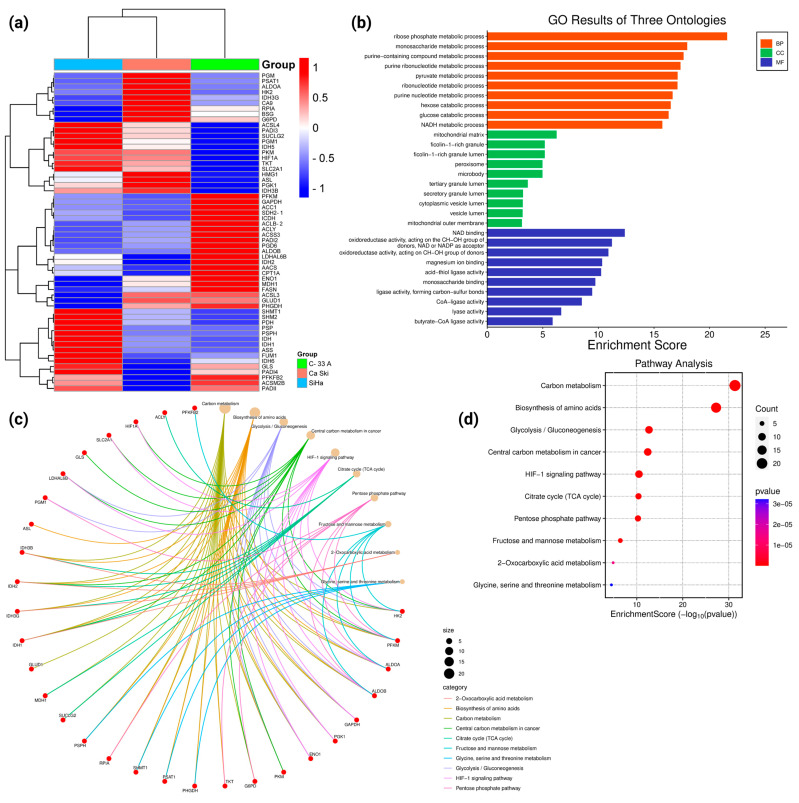
Expression and general biological functions in the cervical cancer cell lines C-33 A (in green), SiHa (in blue), and Ca Ski (in pink). In (**a**) differential gene expression between C-33 A, SiHa, and Ca Ski is depicted in the heat map. Panel (**b**) represents GO analysis from differentially expressed genes in C-33 A, SiHa, and Ca Ski. The colored bars represent the Gene Ontology for molecular functions (MF), molecular components (MC), and biological processes (BP). In (**c**), the cnetplot corresponding to the pathways mainly enriched by genes expressed in C-33 A, SiHa, and Ca Ski is represented. The colored lines correspond to the categories of each pathway, and the genes involved in each enriched pathway are shown. In (**d**), a score dotplot is shown representing the metabolic pathways enriched by genes expressed in C-33 A, SiHa, and Ca Ski cell lines. The size and color of the circles represent the proportion of enrichment. Graphs were obtained from analysis in the KEGG, GO, and GSA databases. The plots were obtained from SRplot. Significant enrichments were defined as |NES| > 1, NOM *p*. adjust < 0.05, and FDR q value < 0.25.

To analyze whether the exometabolome of the cell lines is related to the gene expression of the enzymes responsible for producing the metabolites identified, a gene expression analysis was performed by querying the Affymetrix U133A oligonucleotide microarray GSE9750 dataset deposited in the GEO database (Figure 6a). C-33 A, SiHa, and Ca Ski cell lines were cultured by [21] under conditions similar to those used in our study. The expression of the *PGM, PSAT1, ALDOA, HK2, IDH3G, CA9, RPIA, BSG, HMG1, ASL, PGK1*, and *IDH3B* genes was observed to be higher in the Ca Ski cells than in the C-33 A and SiHa tumor lines. The expression of *ACSL4, PADI3, SUCLG2, PGM1, IDH5, PKM, HIF1A, TKT*, and *SLC2A1* was observed to be higher in the Ca Ski cells than in the C-33 A cells. In contrast, the expression of *ACSL3, GLUD1*, and *PHGDH* is higher in the Ca Ski cells than in the SiHa cells. On the other hand, the expression of *SLC2A1, TKT, HIF1A, PKM, IDH5, PGM1, SUCLG2, PADI3, ACSL4, SHMT1, SHM2, PDH, PSP, PSPH, IDH, IDH1, ASS, FUM1, IDHG, GLS*, and *PADI4* was observed to be higher in the SiHa cells than in the C-33 A and Ca Ski cells. The expression of *HMG1, ASL, PGK1*, and *IDH3B* was found to be higher in the SiHa cells than in the C-33 A cells. In contrast, the expression of *PFKFB2, ACSM2B*, and *PADI1* was higher in the SiHa cells than in the Ca Ski cells. When comparing the expression of the C-33 A cells with those of the SiHa and Ca Ski cells, the expression of the *PFKM, GAPDH, ACC1, SDH2, ICDH, ACLB2, ACCY, ACSS3, PADI2, PGD6, ALDOB, LDHAL6B, IDH2, AACS, CPT1A, ENO1, MDH1*, and *FASN* genes was observed to be higher in the C-33 A cells than in the SiHa and Ca Ski cells. That the expression of *ACSL3, GLUD1*, and *PHGDH* was also observed to be higher in the C-33 A cells than in the SiHa cells, while the expression of *PFKFB2* and *ACSM2B* was higher in the C-33 A cells than in the Ca Ski cells.

In the enrichment analysis of biological pathways and functions performed through the GO and KEGG databases, using the Hypergeometric Test and Gene Set Enrichment Analysis (GSEA) algorithms in both cases, differentially expressed genes (DEGs) were observed in the first part to substantially enrich metabolic processes such as ribose phosphate, monosaccharide, purine-containing compound, purine ribonucleotide, pyruvate, ribonucleotide, purine nucleotide, and NADH metabolic processes, as well as hexose and glucose catabolic processes (Figure 6b). It was observed in the background that DEGs enrich molecular functions such as NAD binding, oxidoreductase activity, acting on the CH-OH group of donors, NAD or NADP as acceptor, magnesium ion binding, acid-thiol ligase activity, monosaccharide binding, ligase activity, forming carbon–sulfur bonds, CoA-ligase activity, lyase activity, butyrate-CoA ligase activity, hydrolase activity, acting on carbon–nitrogen (but not peptide) bonds, and in linear amidines (Figure 6b). Finally, DEGs were observed to enrich the functions of cellular components of the mitochondrial matrix, ficolin-1-rich granule, ficolin-1-rich granule lumen, peroxisome, microbody, tertiary granule lumen, secretory granule lumen, cytoplasmic vesicle lumen, vesicle lumen, and mitochondrial outer membranes (Figure 6b). The pathways enriched in DEGs were the metabolic pathways of carbon metabolism; amino acid biosynthesis; glycolysis/gluconeogenesis; central carbon metabolism in cancer; HIF-1 signaling pathway; the citrate cycle (TCA cycle); the pentose phosphate pathway; fructose and mannose metabolism; 2-oxocarboxylic acid metabolism; and glycine, serine and threonine metabolism (Figure 6c,d).

**Figure 7:**
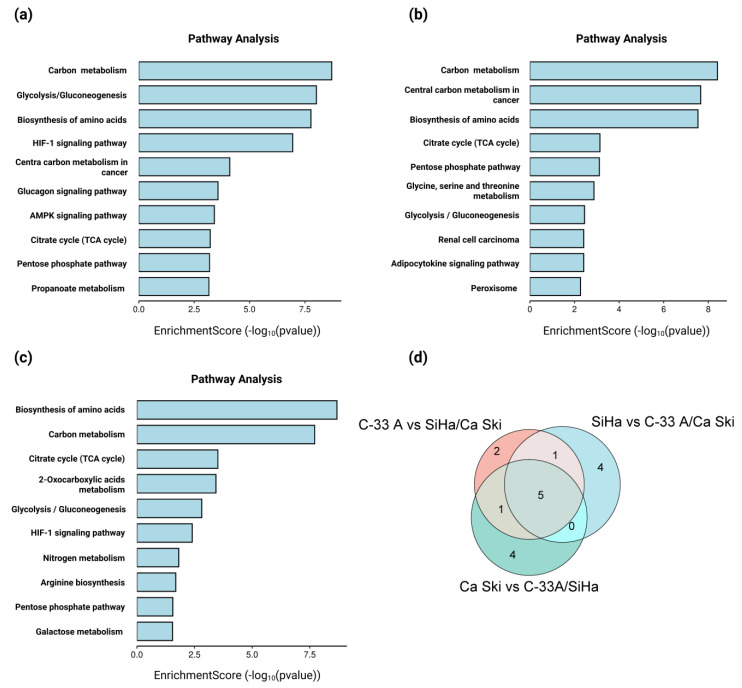
Pathway enrichment analysis of differentially expressed genes in C-33 A, SiHa, and Ca Ski cells. Panels (**a**–**c**) represent the metabolic pathways with the highest enrichment when comparing C-33 A vs. SiHa/Ca Ski, SiHa vs. C-33 A/Ca Ski, and Ca Ski vs. C-33 A/SiHa, respectively. In (**d**), the Venn diagram integrates the commonly enriched pathways in C-33 A vs. SiHa/Ca Ski (pink), SiHa vs. C-33 A/Ca Ski (blue), and Ca Ski vs. C-33 A/SiHa (green). Numbers represent common and specific pathways in the three cell lines. Graphs were obtained from the analysis in the KEGG and GSA databases. Graphs and Ven diagram were obtained from SRplot. Significant enrichments were defined as |NES| > 1, NOM *p*. adjust < 0.05, and FDR q value < 0.25.

We observed that genes expressed in higher proportion in C-33 A cells than in SiHa and Ca Ski cells enrich the metabolic pathways of glucagon signaling and the AMPK signaling pathway (Figure 7a–d); whereas genes overexpressed in SiHa cells as opposed to C-33 A and Ca Ski cells enrich the glycine, serine, and threonine metabolism pathways, adipocytokine signaling pathway, and peroxisome signaling (Figure 7b–d). On the other hand, genes with higher overexpression in Ca Ski cells, as opposed to C-33 A and SiHa cells, were observed to enrich the pathways of 2-oxocarboxylic acid metabolism, nitrogen metabolism, arginine biosynthesis, and galactose metabolism (Figure 7c,d). In the three models, the genes with the highest expression were observed to enrich pathways related to carbon metabolism, glycolysis/gluconeogenesis, amino acid biosynthesis, the tricarboxylic acid cycle (TCA cycle), and the pentose phosphate pathway (Figure 7d).

**Figure 8:**
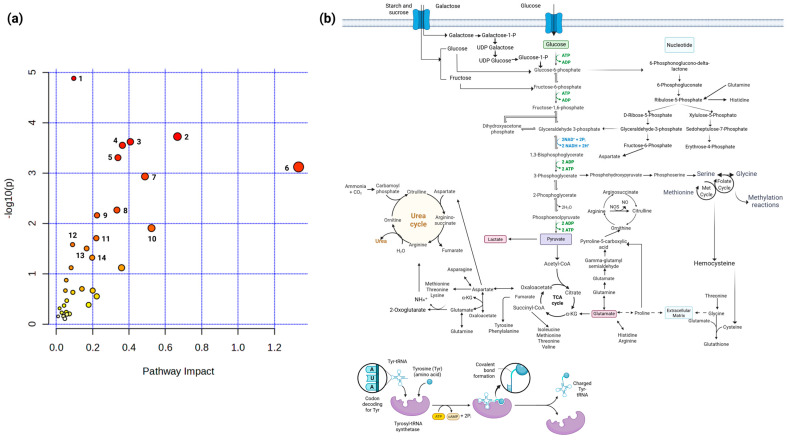
Metabolic pathways favored by the effect of HPV 16. (**a**) Enrichment of metabolic pathways favored by HPV 16. The overview shows all matching pathways according to the *p*-values from the pathway enrichment analysis and the impact values from the pathway topology analysis. Each node represents a metabolic pathway, nodes colored red and numbered 1 to 14 represent metabolic pathways with a *p*-value > 0.05. (1) Aminoacyl-tRNA biosynthesis; (2) Pentose phosphate pathway; (3) Arginine biosynthesis; (4) Valine, leucine and isoleucine biosynthesis; (5) Glycolysis or Gluconeogenesis; (6) Neomycin, kanamycin and gentamicin biosynthesis; (7) Citrate cycle (TCA cycle); (8) Nitrogen metabolism; (9) Glycine, serine and threonine metabolism; (10) Starch and sucrose metabolism; (11) Galactose metabolism; (12) Glutathione metabolism; (13) Alanine, aspartate and glutamate metabolism and (14) One carbon pool by folate. (**b**) The integration of the metabolic pathways favored by HPV 16 is shown. The diagram was constructed by synthesizing the metabolic pathways presented in panel (**a**), and each pathway represented by a node was retrieved from the KEGG database.

The dataset of metabolites and genes with increased concentration and expression, respectively, in HPV 16-positive and -negative cell lines was integrated to obtain the metabolic pathways favored by the effect of HPV 16. The analysis was performed using the MetaboAnalyst 6.0 web platform, which allows for the integration of metabolomic data, their interpretation, and their connection with other omics data from statistical and functional perspectives [22]. The analysis was performed using the joint pathway analysis option, through tight integration, which combines the list of metabolites and the list of significant genes with optional fold change values. For the enrichment analysis, the hypergeometric test was applied; for the topology measure, the degree centrality was applied; and for the integration method, a combination of queries was performed. We observed that gene overexpression and metabolite concentration (Figure 8) significantly favor (*p*-value > 0.05) the enrichment of the metabolic pathways of aminoacyl-tRNA biosynthesis; pentose phosphate pathway; arginine biosynthesis; valine, leucine, and isoleucine biosynthesis; glycolysis or gluconeogenesis; neomycin, kanamycin, and gentamicin biosynthesis; citrate cycle (TCA cycle); nitrogen metabolism; glycine, serine, and threonine metabolism; starch and sucrose metabolism; galactose metabolism; glutathione metabolism; alanine, aspartate, and glutamate metabolism; and one carbon pool by folate, suggesting that the presence and copy number of HPV 16 promote the production of energy and biomass synthesis necessary for tumor progression and maintenance in CC.

## 3. Discussion

The extraordinary versatility of cancer cells to reprogram their metabolism to meet energy demands, even in the presence of oxygen, was first described almost a century ago by Otto Warburg [23]. This premise has opened the door for the study of cancer metabolism. It is now known that not only are energy requirements essential in the study of cancer, but also that the a posteriori metabolic activity of tumors can be exploited as an analytical advantage. A large number of biologically active products from tumor metabolism are secreted into the tumor microenvironment, especially under conditions of unbalanced growth, and have been proposed as potential cancer-specific exometabolomic fingerprints [24]. Even though metabolomics studies in CC focus on characterizing and quantifying metabolic processes within the cell, metabolic differences in CC patients have been identified, ranging from the vaginal microenvironment to CC subtypes, the presence of HR-HPV, and the effectiveness of radiotherapy and chemotherapy treatments [20,25,26,27]. This suggests that specific metabolites may provide important insights into the mechanisms involved in tumor development and progression and could be potential candidates for new biomarkers of disease progression, early diagnosis, and treatment [25].

Exometabolomics is the study of how cells transform their environment via small molecules in culture media as a result of cell culture, typically by comparing media with different cellular conditions. Exometabolomics provides powerful insights into cellular phenotypes while circumventing the challenges of measuring intracellular metabolites, which are known to be altered and degraded within milliseconds of extraction [19,28]. This work focused on identifying potential biomarkers of CC diagnosis and progression by analyzing the exometabolomic profile using ^1^H NMR of three CC cell models with significant molecular differences: the C-33 A cell line (HPV-negative), SiHa cells (with one to two copies of HPV 16 DNA integrated into their genome), and Ca Ski cells (with about 600 copies of HPV 16 DNA in a mixed, genome-integrated, and episomal form) [29].

According to the analysis of the cell growth medium via ^1^H NMR, the three cell types were found to have unique exometabolomic profiles. Following discriminant model analysis, a marked significant difference observed not only between the metabolomic profiles of HPV-negative CC cells (C-33 A) and HPV-positive cells (SiHa and Ca Ski) but also between HPV 16-positive cells themselves. It is known that infection with human papillomavirus (HPV), particularly high-risk (HR) HPV types such as HPV 16, is an important factor in the progression of cervical cancer (CC) [30,31] and that the E6 and E7 oncoproteins of HPV 16 may promote metabolic reprogramming in CC. For example, E6 (via p53 and HIF-1α) and E7 (via pRb) increase anaerobic glycolysis even in the presence of oxygen and graded acid synthesis [16,17]. Proliferating cells must de novo synthesize several nitrogen-containing molecules, including nucleotides, non-essential amino acids, and polyamines [32,33]. When the exometabolomic profile of C-33 A cells was compared with the SiHa cell line, significant differences in glutamine and uracil concentrations were observed. When comparing the exometabolomic profile between the Ca Ski cell line against C-33 A and SiHa cells, the concentration of metabolites, such as glutamine and uracil, was observed to be higher in the Ca Ski cell line compared to the C-33 A and SiHa cells. However, when comparing the concentration of metabolites in HPV 16-positive and -negative cell lines, leucine, isoleucine, valine, lysine, methionine, glutamine, ornithine, choline, glucose, and tryptophan showed statistically significant differences.

Pyrimidines interact with biological macromolecules, such as receptors and enzymes, which are active components of RNA. Among these components, uracil is widely used for the synthesis of biologically relevant compounds in tumor cells [34]. The ketone body 3-hydroxybutyrate was observed to be elevated in ovarian cancer and to be associated with cancer cell invasion and migration [35,36]. Glutamine and glucose are used as building blocks for the assembly of various macromolecules and are considered critical molecules for tumor growth [37]. Glutamine is transformed into glutamate, which promotes TCA anaplerosis to ensure ATP maintenance.

Furthermore, glutamate stimulates the uptake of cysteine, which is then used as an antiporter and expelled into the extracellular milieu. Glutamine also participates in the incorporation of leucine, valine, tyrosine, and phenylalanine, as well as in the creation of substrates for glycosylation reactions and in the synthesis of heparan sulfate [37]. Acetyl-CoA has been reported to possibly be produced from lysine catabolism. Thus, acetyl-CoA can function as an obligate substrate for enzymes that acetylate histones and other proteins, mediating the deposition and removal of epigenetic marks from chromatin [38]. The metabolic state of a cancer cell may not only affect its long-term decision making, but also has the potential to influence the fate of neighboring cells [39]. Cancer cells often face nutrient-poor environments and develop various strategies for utilizing metabolic intermediates to circumvent these limitations, as has been described with metabolite secretion, where metabolites secreted into the extracellular milieu are reused by neighboring cells to enhance further cancer cell metabolism, which actively contributes to the emergence of a more aggressive phenotype [37]. These data suggest that the absence or presence (as well as the density) of HPV 16 DNA integration into cell genomes is key to each metabolomic profile identified, and that changes in exometabolomic profiles may contribute to the persistence of the virus and the process by which HPV 16 infection induces CC [27].

To date, our study is the first to describe the exometabolomic profiles of CC cells, and this comprehensive and specific approach could be useful in discovering new biomarkers for disease diagnosis and prognosis. HPV 16 oncoproteins E6 and E7 are known to modulate exometabolism by increasing the expression of genes that direct metabolic processes [40], including glucose metabolism, fatty acid synthesis, and glutamine metabolism [17,41,42]. Here, using a transcriptomic approach, we validate and correlate the exometabolomic profiles with those from the GSE9750 dataset obtained from the GEO repository; an overexpression of metabolic enzymes was obtained in HPV 16-positive and -negative CC cell lines. We observed that the expression of *PGM*, *PSAT1*, *ALDOA*, *HK2*, *IDH3G*, *CA9*, *RPIA*, *BSG*, *HMG1*, *ASL*, *PGK1*, and *IDH3B* is higher in Ca Ski cells compared with in SiHa and C-33 A cells. When comparing the expression of SiHa cells against Ca Ski and C-33 A cells, we observed that the expression of *SLC2A1*, *TKT*, *HIF1A*, *PKM*, *IDH5*, *PGM1*, *SUCLG2*, *PADI3*, *ACSL4*, *SHMT1*, *SHM2*, *PDH*, *PSP*, *PSPH*, *IDH*, *IDH1*, *ASS*, *FUM1*, *IDHG*, *GLS* and *PADI4* genes is higher in SiHa cells, while the expression of *PFKM*, *GAPDH*, *ACC1*, *SDH2*, *ICDH*, *ACLB2*, *ACCY*, *ACSS3*, *PADI2*, *PGD6*, *ALDOB*, *LDHAL6B*, *IDH2*, *AACS*, *CPT1A*, *ENO1*, *MDH1*, and *FASN* is higher in C-33 A cells than in Ca Ski and SiHa cells. We also observed that gene overexpression in Ca Ski cells, compared with in C-33 A and SiHa cells, favored the enrichment of amino acid biosynthesis pathways; carbon metabolism, including the TCA cycle; glycolytic metabolism; gluconeogenesis; nitrogen metabolism; arginine biosynthesis; the pentose phosphate pathway; and galactose metabolism. These validated data are in agreement with the pathways that are mostly enriched when relating overexpressed genes and metabolites with higher concentrations of Ca Ski cells. In addition to the pathways mentioned above, pyruvate metabolism and the HIF-1α transcription factor signaling pathway were observed to gain importance in this analysis. It is known that high levels of HIF-1α can be associated with a predictive biomarker of CC [41].

Moreover, in CC, HIF-1α favors reprogramming to glycolytic metabolism and decreases TCA metabolism, as well as oxidative phosphorylation, through an increase in pyruvate concentration, which serves as an intermediate for lactate production [10,42,43,44]. The increase in the TCA pathway has also been suggested to be mediated by amino acids, such as glutamine, and this mechanism has been suggested to favor the production of TCA intermediates for the synthesis of cholesterol and fatty acids [10]. We consider that the high concentration of glutamine excreted by Ca Ski cells may favor TCA metabolism in the cells that comprise the tumor microenvironment, thereby balancing the acquisition of nutrients and biomolecules necessary for tumor maintenance.

On the other hand, the concentration of glucose could be even more inclined to activate the pentose phosphate pathway and consequently to obtain pyrimidine precursors such as uracil [10,34]. The genes overexpressed in HPV 16 cells were observed to favor arginine biosynthesis and to be in line with the metabolites observed in the exometabolomic analysis that favor its synthesis, such as ornithine [45]. Arginine has also been reported to possibly favor cancer progression and maintenance [45] through its epigenetic modifications, which ensure cell survival by regulating epigenetic-mediated gene expression, mRNA splicing, and response to DNA damage [46]. Thus, arginine could be considered an important biomarker in the treatment and diagnosis of CC, as reported in previous studies [45]. These data indicate that the presence and copy number of HPV 16 ensure viral persistence, energy production, and biomass synthesis, which are necessary for tumor progression and maintenance in CC and, consequently, the death of cancer patients.

CC is a serious public health problem, and although vaccination and screening programs have significantly reduced the incidence of CC worldwide by up to >80%, the mortality rate in low-income countries remains high. The search for predictive biomarkers to identify patients at risk of developing CC in a timely manner remains a limitation [47]. In this study, an exploratory exometabolomic approach was used with the primary objective of characterizing the differential metabolomic profile and its relationship with gene expression between HPV 16-positive and -negative cervical cancer (CC) cells. This approach allowed us to preliminarily identify potentially biologically relevant candidate metabolites, which could be validated in future studies with larger sample sizes. However, these findings should be interpreted with caution until confirmed in independent cohorts. Despite this limitation, this initial approach suggests potential diagnostic and prognostic biomarkers, as well as therapeutic targets, in HPV 16-associated CC.

## 4. Materials and Methods

### 4.1. Workflow Diagram

**Figure 9:**
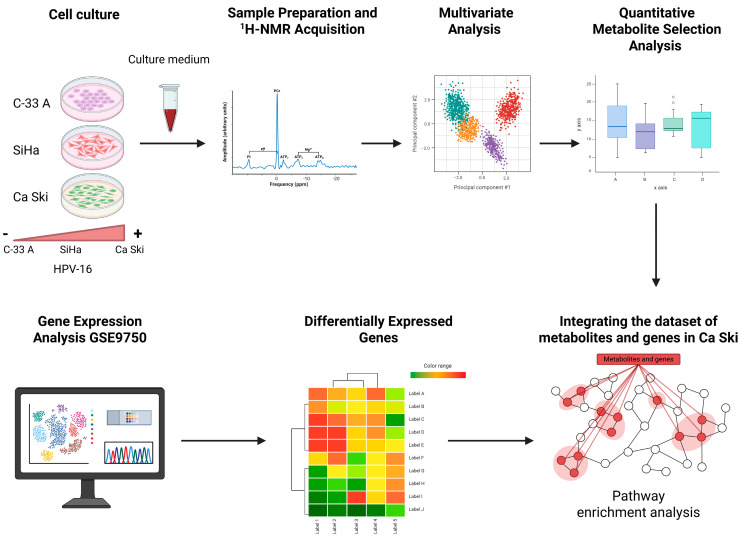
The figure represents the sequential graph of the steps, decisions and processes of a workflow.

The procedure carried out in this work is summarized below in a step-by-step chart (Figure 9).

### 4.2. Cell Culture

The CC cell lines C-33 A HTB-31™ (negative for human papillomavirus DNA and RNA), SiHa HTB-35™ (HPV 16 positive; 1 to 2 copies of viral DNA integrated into the cell genome), and Ca Ski CRL-1550™ (HPV 16 positive, with approximately 600 copies of viral DNA integrated into the cell genome) were acquired from the ATCC and were cultured in DMEM (Dulbecco’s modified Eagle’s medium) supplemented with 10% fetal bovine serum (Gibco, Life Technologies, Grand Island, NY, USA), and 100 U/mL of penicillin and 100 µg/mL of streptomycin (Gibco, Life Technologies, Grand Island, NY, USA) were added. The cells were maintained at 37 °C in a 5% CO_2_ atmosphere.

### 4.3. Sample Preparation and ^1^H-NMR Acquisition

Metabolomic profiles were obtained using ^1^H-NMR. The culture medium was transferred to an Eppendorf tube and centrifuged to recover the supernatant, which was frozen in liquid nitrogen and stored at −80 °C until use. At the time of NMR analysis, the samples were thawed on ice. A total of 10% D_2_O (5 mM TSP, 140 mM Na_2_HPO_4_, 0.04% NaN_3_, pH 7.4) was added and then transferred to an NMR tube to determine the metabolomic profiles using a Bruker Avance II 600 MHz spectrometer (Bruker, Billerica, MA, USA) at 310 K (37 °C) [48,49]. The assignment of the identified metabolites to the exometabolome was determined using Amix v3.9.7 in combination with the Bruker BBIOREFCODE 2.0.0 NMR metabolic profiling database (Bruker Biospin, Rheinstetten, Germany), as well as other existing public databases and literature reports [50,51].

### 4.4. Multivariate Analysis

^1^H NMR spectra were integrated using Amix 3.9.7 (Bruker Biospin, Rheinstetten, Germany) in the δ 9.00–0.80 ppm region. The region corresponding to the residual water signal (δ 5.06–4.30 ppm) was excluded from the analysis to avoid interferences arising from differences in water suppression. All intensities from the integrated regions were normalized based on the protein quantity determined in the corresponding samples. The generated intensity tables were imported into SIMCA-P 12.0 software (Umetrics AB, Umeå, Sweden). Before statistical analysis, the data were Pareto-scaled.

PCA was used to examine intrinsic variability within the dataset, to observe clustering or separation trends, and to identify outliers. OPLS-DA was applied to minimize the potential contribution of intergroup variability and to improve the separation between sample groups further. The Centered and scaled regression coefficients (CoeffCS) and their Jack-knife standard error of the coefficients (CoeffCScvSE) computed from all rounds of cross validations were used to discard spectral regions that were redundant or not correlated to the response. With this strategy, only those variables, with a ratio between CoeffCS and CoeffCScvSE > 1, were included in the model. Once applied, the variable selection method, a new OPLS-DA model, was calculated and internal cross validation and permutation tests were performed to evaluate the model obtained. The default 7-fold internal cross-validation method was applied, from which the Q^2^ (predictive ability parameter, estimated by cross-validation) and R^2^X (goodness-of-fit parameter) values were extracted. These parameters, the VIP criteria > 1, *p* < 0.05 along with the corresponding permutation tests (n = 100), were used to assess the quality of the obtained OPLS-DA models. SUS plots were also obtained to assess shared (metabolites aligned with the diagonals) and unique (metabolites aligned with the axes) differences between the two OPLS-DA statistical models.

### 4.5. Quantitative Metabolite Selection Analysis

The main metabolites contributing to group discrimination in each model were integrated using Amix 3.9.7. The normality of the distribution of variables was assessed using Student’s *t*-test. A *p*-value < 0.05 (95% confidence level) was considered statistically significant.

### 4.6. Gene Expression Analysis

Gene expression analysis was performed using the public functional genomics data repository Gene Expression Omnibus (GEO) (https://www.ncbi.nlm.nih.gov/geo/), which provides tools to help users query and download selected experiments and gene expression profiles [52]. Gene expression profiles of the C-33 A, SiHa, and CaSki cell lines were compared using the GSE9750 dataset (Affymetrix U133A oligonucleotide microarray (Santa Clara, CA, USA)) [21].

### 4.7. GO, KEGG and GSEA of Differentially Expressed Genes in C-33 A, SiHa, and Ca Ski Cells

Enrichment analysis was performed using the SRplot web server (https://www.bioinformatics.com.cn/srplot) with the access date on 9 January 2025. SRplot integrates all commonly used data visualization and plotting functions with over 2700 citations [53]. The Gene Ontology (GO) database (http://geneontology.org/), the Kyoto Encyclopedia of Genes and Genomes (KEGG) database (http://www.kegg.jp/kegg/kegg1.html (accessed on 25 June 2025) and the Gene Set Enrichment Analysis (GSEA) database (https://www.gsea-msigdb.org/gsea (accessed on 30 June 2025)) were employed for pathway enrichment analysis of differentially expressed genes, which facilitated the annotation of their biological functions and molecular mechanisms. To differentiate the enriched and common pathways in the different cellular models, Venn diagrams were created using the Venny 2.0 web server (https://bioinfogp.cnb.csic.es/tools/venny/index2.0.2.html (accessed on 30 June 2025)). Significant enrichment parameters were defined as |NES|>1, NOM *p*.adjust < 0.05, and FDR q value < 0.25. The metabolic pathways enriched by the effect of HPV 16 were created by integrating the dataset of metabolites and genes that increase their concentration and expression, respectively, in the Ca Ski cell lines. The analysis was performed on the MetaboAnalyst 6.0 web platform, which allows the integration of metabolomic data, their interpretation, and integration with other omic data from a statistical and functional context (https://www.metaboanalyst.ca/MetaboAnalyst/home.xhtml (accessed on 14 July 2025)) [22]. The pathways with the highest enrichment were defined based on the Hits and *p*-value.

### 4.8. Statistical Analysis of Gene Expression

Gene expression of the C-33 A, SiHa, and CaSki cell lines was analyzed using the GSE9750 dataset (Affymetrix U133A oligonucleotide microarray (Santa Clara, CA, USA)). Gene expression data were calculated from data processed by Scotto et al. [21]. Signal intensity values were obtained using the perfect match/mismatch (PM/MM) difference model, followed by within-chip and between-chip signal normalization using model-based expression. Data were processed using the SRplot web server (https://www.bioinformatics.com.cn/srplot) (accessed on 20 July 2025)). Default parameters from several databases were used for statistical analysis in this study. Differential data were visualized using the ggplot2 package. Statistical differences in metabolite intensities were calculated using Student’s *t*-test. A * *p* < 0.05, ** *p* < 0.01, *** *p* < 0.001 value was used.

## 5. Conclusions

In conclusion, this study demonstrated the impacts of HPV 16 on the exometabolomic profile and gene expression patterns of CC cell lines. Our findings indicate that HPV 16 infection and its integrated copy number differentially deregulate cellular processes, resulting in distinct exometabolomic and transcriptomic signatures. HPV 16-positive cell lines exhibited elevated concentrations of leucine, isoleucine, valine, lysine, methionine, glutamine, ornithine, choline, glucose, and tryptophan. These metabolic alterations were associated with the upregulation of genes involved in metabolic reprogramming, including aminoacyl-tRNA biosynthesis, the pentose phosphate pathway, amino acid metabolism, glycolysis, the tricarboxylic acid cycle, nitrogen metabolism, starch and sucrose metabolism, galactose metabolism, glutathione metabolism, aspartate and glutamate metabolism, and nucleotide biosynthesis through the folate-mediated one-carbon pool. Collectively, these findings highlight metabolites and metabolism-associated genes that may serve as robust, non-invasive diagnostic and prognostic biomarkers, as well as potential therapeutic targets for HPV 16-related CC. Furthermore, these insights may be extended to the evaluation, monitoring, and therapeutic response of patients with CC.

## Figures and Tables

**Table 1:** Intensities and significant variations in metabolites were found in the culture medium where C-33 A, SiHa, and Ca Ski cell lines were cultured.

Metabolite	Integration Area	*T*-Test SiHa vs. CaSki	*T*-Test C-33 A vs. SiHa	*T*-Test C-33 A vs. CaSki	*T*-Test HPV− vs. HPV+
Leucine	0.9442–0.9676	0.2407	0.0679	0.1018	0.0204 *
Isoleucine	0.9889–1.0137	0.2556	0.0772	0.1214	0.0269 *
Valine	1.0141–1.0419	0.2849	0.1150	0.1468	0.0313 *
3-hydroxybutyrate	1.1986–1.2222	0.3157	0.3516	0.8938	0.5077
3-hydroxyisovalerate	1.2505–1.2595	0.1539	0.8354	0.1721	0.3751
Alanine	1.4511–1.4935	0.3036	0.1459	0.2276	0.0724
Lysine	1.6981–1.7369	0.2092	0.1081	0.2252	0.0458 *
Acetic acid	1.8904–1.9226	0.2193	0.6337	0.1897	0.3280
Methionine	2.1293–2.1333	0.1189	0.0584	0.1362	0.0296 *
Glutamate	2.3255–2.3534	0.2576	0.2503	0.9440	0.2456
Pyruvate	2.3586–2.3681	0.2742	0.2559	0.7578	0.2140
Glutamine	2.4254–2.4571	0.0153 *	0.0039 **	0.2779	0.0358 *
Citrate	2.6594–2.6741	0.3286	0.3681	0.8566	0.4766
2-oxoglutarate	2.9752–3.0056	0.2067	0.2635	0.4724	0.3942
Ornithine	3.0491–3.0755	0.9719	0.2148	0.0946	0.0217*
Choline	3.1807–3.1953	0.1825	0.1000	0.1130	0.0364 *
Glycine	3.546–3.5583	0.3019	0.1619	0.2208	0.0536
Lactate	4.0728–4.133	0.2313	0.3054	0.3710	0.4316
Pyroglutamate	4.142–4.187	0.2502	0.2899	0.7799	0.4000
Threonine	4.2218–4.2753	0.1645	0.1909	0.7717	0.3239
Glucose	5.2005–5.2506	0.1387	0.2339	0.0288 *	0.0498 *
Uracil	5.7844–5.8088	0.4696	0.0462 *	0.0773	0.0637
Fumarate	6.5–6.5142	0.3342	0.2578	0.4848	0.1814
Tyrosine	6.8701–6.9202	0.2801	0.1660	0.2971	0.0771
Histidine	7.0391–7.1008	0.3013	0.1875	0.3953	0.1051
Phenylalanine	7.3529–7.443	0.2571	0.1543	0.2425	0.0647
Tryptophan	7.7141–7.741	0.3551	0.1391	0.1629	0.0390 *
Formate	8.4346–8.4565	0.4352	0.2927	0.3567	0.1388
Nicotinate	8.589–8.6114	0.5253	0.5427	0.1556	0.2038

Bold letters highlight metabolites with significant differences when compared between cell groups using Student’s *t* tests. Metabolites with significant differences were obtained by multiple comparison of the FDR between cell line culture media. Statistical significance: * *p* < 0.05, ** *p* < 0.01.

## Data Availability

The links to the expression data were posted in the Section 4.

## Abbreviations

The following abbreviations are used in this manuscript:

CC	Cervical cancer
HPV 16	Human papillomavirus 16
HPV-HR	High-risk HPVs
^1^H-NMR	^1^H nuclear magnetic resonance
VHL	von Hippel–Lindau
GEO	Gene Expression Omnibus
HIF-1α	Hypoxia-inducible factor-1α
KEGG	Kyoto Encyclopedia of Genes and Genomes
PCA	Principal component analysis
GO	Gene Ontology
OPLS-DA	Orthogonal Partial Least Squares Discriminant Analysis
PGM	Phosphoglucomutase
PSAT1	Phosphoserine Aminotransferase 1
ALDOA, B	Aldolase A and B
HK2	Hexokinase 2
IDH3G	Isocitrate Dehydrogenase (NAD(+)) 3 Non-Catalytic Subunit Gamma
CA9	Carbonic Anhydrase 9
RPIA	Ribose 5-Phosphate Isomerase A
BSG	Basigin
HMG1	High Mobility Group Box 1
ASL	Argininosuccinate Lyase
PGK1	Phosphoglycerate Kinase 1
IDH3B	Isocitrate Dehydrogenase (NAD(+)) 3 Non-Catalytic Subunit Beta
ACSL3, 4	Acyl-CoA Synthetase Long-Chain Family Member 3 and 4
PADI2, 3, 4	Peptidyl Arginine Deiminase 2, 3 and 4
SUCLG2	Succinate-CoA Ligase GDP-Forming Subunit Beta
PGM1	Phosphoglucomutase 1
IDH1, 2 and 5	Isocitrate Dehydrogenase 1, 2 and 5
PKM	Pyruvate Kinase M1/2
HIF1A	Hypoxia Inducible Factor 1 Subunit Alpha
TKT	Transketolase
SLC2A1	Solute Carrier Family 2 Member 1
GLUD1	Glutamate Dehydrogenase 1
PHGDH	Phosphoglycerate Dehydrogenase
SHMT1	Serine Hydroxymethyltransferase 1
SHM2	Serine Hydroxymethyltransferase 2
PDH	Pyruvate Dehydrogenase
PSPH	Phosphoserine Phosphatase
ASS	Argininosuccinate Synthase
FUM1	Fumarate hydratase 1
IDHG	Isocitrate Dehydrogenase Gamma
GLS	Glutaminase
HMG1	High Mobility Group 1
PFKFB2	6-Phosphofructo-2-Kinase/Fructose-2,6-Biphosphatase 2
ACSM2B	Acyl-CoA Synthetase Medium-Chain Family Member 2B
PADI1	Peptidyl Arginine Deiminase 1
PFKM	Phosphofructokinase, Muscle
GAPDH	Glyceraldehyde-3-Phosphate Dehydrogenase
ACC1	Acetyl-CoA carboxylase 1
SDH2	Succinate Dehydrogenase 2 or B
ICDH	Isocitrate Dehydrogenase
ACLB2	ATP-citrate synthase beta chain protein 2
ACCY	Pyruvate oxidase/decarboxylase
ACSS3	Acyl-CoA Synthetase Short Chain Family Member 3
PGD6	phosphogluconate dehydrogenase 6
LDHAL6B	Lactate Dehydrogenase A-Like 6B
AACS	Acetoacetyl-CoA Synthetase
CPT1A	Carnitine Palmitoyltransferase 1A
ENO1	Enolase 1
MDH1	Malate Dehydrogenase 1
FASN	Fatty Acid Synthase
